# Alcoholic Drinks and Phthisis

**Published:** 1861-09

**Authors:** 


### ALCOHOLIC DRINKS AND PHTHISIS.

From a paper by Prof. Austin Flint, in the *Am. Jour. Med. Sci.*, we extract the following judicious observations:

It is desirable, then, to inquire whether the statistical results given in the report by Prof. Davis warrant the conclusions which he draws from them. We propose to raise this inquiry.

Let us see, in the first place, whether Prof. Davis has presented all the postulates involved in the problem which he undertakes to solve. To determine the influence of alcoholic drinks on the development of phthisis, it is not enough to ascertain that a certain proportion of patients affected with this disease has been addicted to the use of these drinks. No one will assert that the use of alcoholic drinks affords an infaillible protection against the disease. The question is, whether they who habitually use these drinks are rendered thereby less liable to this disease than they would otherwise be. Now, it is ascertained that a certain proportion of a given number of patients affected with phthisis have been accustomed to use alcoholic drinks. This is one point in the problem. Another essential point is to ascertain whether this proportion falls below or exceeds the proportion who, in like manner, have used these drinks out of an equal number of non tuberculous persons in similar conditions of life. It is plain that we must have some standard of comparison by which to judge whether the proportion of drinkers among patients affected with phthisis be so large as to show an antagonizing influence of alcoholic drinks, or so small as to lead to an opposite conclusion. Prof. Davis appears not to have considered the importance of this in assuming that the results of the analysis of his cases leads to the conclusion which he draws from them. In fact, the statistical results of his analysis constitute but a preliminary step to the inquiry concerning the influence of alcoholic drinks on the development of pulmonary tuberculosis. Having obtained these results, the next point to be settled is, whether they show among tuberculous patients a large or small ratio of those who are addicted to the use of alcoholic drinks. How is this point to be settled? Plainly, the only way is to ascertain the ratio among patients not tuberculous, from the same classes of society of those who are equally addicted to the use of alcoholic drinks, and then institute a comparison of the results of the two analysis. Whether the results of Prof. Davis's analysis prove the influence of alcoholic drinks to be favorable to the development of phthisis, or otherwise, is thus, as we conceive, a problem to be settled by facts to which Prof. Davis makes no reference. In the absence of these facts, we have as much right to assume that the statistics given by Prof. Davis prove the prophylactic influence of alcoholic drinks, as he has to assume an opposite conclusion. Without assuming the former, we believe it will be found to be true.

The cases recorded by Prof. Davis occurred in hospital and private practice. He does not state the proportion of hospital cases, but we infer that they constitute the large majority, from the fact that, of the 210 cases, in all but sixty the patients were foreigners, and the author indeed gives, as an explanation of the large number of patients from Ireland (85), the fact of his being brought into connection with this class in the Mercy Hospital of Chicago. We will suppose all the 210 cases to have been hospital cases. In 51 of these 210 cases, the patients had wholly abstained from the use of alcoholic drinks. This is certainly a large proportion of persons practising total abstinence to be found among the classes of patients received into a charity hospital. Would there be found 51 persons who had practised total abstinence for years among 210 hospital patients not affected with tuberculosis? But of the remaining 159 cases, in 91 the patients only used alcoholic drinks occasionally, some very sparingly and only on festive occasions. Now, it is absurd to suppose that an occasional indulgence, whether sparingly or in excess, is of any appreciable account in determining the influence of alcoholic drinks on the development of phthisis. These cases are to be excluded. So far as any influence is to be attributed to alcoholic drinks, the class of cases only is to be considered in which the persons "had used some form of alcoholic beverage almost daily from one to twelve years previous to the active signs of tuberculosis." To this class belong 68 of the 210 cases, or a fraction over 32 per cent. Now, is this a large or a small per centage? This question is to be answered after ascertaining the percentage of a similar class, as regards the use of alcoholic drinks, in 210 hospital cases not tuberculous. We are not in a situation, at the present moment, to appeal to facts, but we shall be surprised if they do not show a larger percentage; if so, assuming that Prof. Davis's cases were mostly hospital cases, his statistics will clearly prove the reverse of the inference which he deduces from them. Taking the statistics as they stand, we may reason *for* a prophylactic influence of alcoholic drinks precisely as Prof. Davis does *against* such an influence, and, as we conceive, with greater force. Prof. Davis's argument is, so large a proportion as 68 of 210 tuberculous patients being habitual drinkers, it is proved that alcoholic drinks exert no influence to prevent phthisis. *Per contra*, it may be said, so large a proportion as 142 of 210 tuberculous patients not being habitual drinkers, it is proved that alcoholic drinks do exert a positive influence in preventing phthisis.

With regard to the value of alcohol as a remedy in pulmonary tuberculosis, Prof. Davis admits that an apparent improvement may be produced by it, but he says, "Truth compels me to say that I have never seen a case in which this apparent improvement was permanent." Inasmuch, however, as he states that the idea of alcoholic beverages preventing the development and retarding the progress of this disease when it was announced, was not in accordance with his views, and he began to collect cases in disapproval of this idea as long ago as 1855, it is fair to presume that he has not employed alcohol in the treatment of this disease to much extent. We confess ourselves to be among those who have been laid by clinical observation to attach no small value to alcoholic beverages in the treatment of phthisis. We are certainly able to cite a number of cases in which we have seen not merely a temporary, but a permanent improvement under their use, in conjunction with hygienic influences. But we reserve the consideration of this subject for some other occasion. * * * *

### DE WITT COUNTY MEDICAL SOCIETY.

The Society met in the office of Dr. R. T. Richards, in Mt. Pleasant, Oct. 1st, 1861.

The meeting was called to order by the President at 10 o'clock A. M. The minutes of the previous meeting were read and approved.

Dr. R. T. Richards proposed Dr. J. R. Richards as a candidate for membership.

Society adjourned to meet at 2 o'clock P. M.

### AFTERNOON SESSION.

The Censors, having examined the applicant, Dr. J. R. Richards, reported favorable to his election. Dr. Richards was then elected a member of the Society.

At a previous meeting of the Society, held in Marion, the first Tuesday in July last, Drs. Norris and Fairchild were expelled from the Society; but in order to give them an opportnnity to come up and defend themselves, that portion of the minutes were not published. As they have not come up, therefore, the Society has resolved to sustain the proceedings of the previous meeting, and has ordered the Secretary to publish that portion of the minutes that were not published before, which is as follows:

Dr. Tyler charged Dr. Norris with unprofessional conduct.

Dr. Madden charged Dr. Fairchild with ungentlemanly, immoral, and unprofessional conduct. Drs. Norris and Fairchild were expelled from the Society.

On motion of Dr. Shurtleff, Dr. Waters was invited to participate in the meeting.

Essays being in order, Dr. R. T. Richards read an excellent essay on Typhoid Fever. The symptoms, pathology and treatment was in accordance with the general received opinions of the present day. After the reading of the essay there was a general discussion in reference to Typhoid fever.

Dr. Wright also read an essay, on Diphtheria, which was also followed by a lengthy discussion. Some regarded Diphtheria as being, primarily, a local disease; but nearly all looked upon it as being a disease of the whole system—the throat affection being only one of its local manifestations, or expressions.

Dr. Tyler offered the following resolutions, which were adopted:

*Resolved*, That a vote of thanks is due and is hereby tendered to Drs. Wright and Richards for their able essays read on the present occasion.

*Resolved*, That a vote of thanks be tendered to Dr. R. T. Richards, for the excellent dinner served up at the hotel for the members of this Society.

The President announced Pneumonia as the subject for general discussion at the next meeting.

Drs. Shurleff and J. R. Richards were appointed to read essays. Drs. Edmiston and Adams were continued.

On motion, the proceedings of this meeting were ordered to be published in the Central *Transcript* and Chicago MEDICAL JOURNAL.

On motion, the Society adjourned, to meet at Waupella the first Tuesday in January next, at 10 o'clock A. M.

JOHN WRIGHT, M. D., Secretary, *pro tem.*

